# Amino Acids Supplementation in Cancer: What Do We Feed, the Patient or the Tumor?

**DOI:** 10.3390/nu17172813

**Published:** 2025-08-29

**Authors:** Giovanni Corsetti, Evasio Pasini, Claudia Romano, Francesco S. Dioguardi

**Affiliations:** 1 Department of Clinical and Experimental Sciences, University of Brescia, 25128 Brescia, Italy; evpasini@gmail.com (E.P.); cla300482@gmail.com (C.R.); 2 Institute of Human Health, Ruaha Catholic University, Iringa P.O. Box 774, Tanzania; 3 Nutri-Research s.r.l., 20127 Milan, Italy; fsdioguardi@gmail.com

**Keywords:** cancer, amino acids, malnutrition, sarcopenia, mTOR, food integration

## Abstract

Background/Objectives: Diet and obesity contribute to approximately 50% of tumor development. Therefore, nutrition plays a key role not only in cancer prevention but also in determining prognosis. Notably, between 30% and 90% of cancer patients experience malnutrition. Furthermore, the hypercatabolic state induced by tumors leads to widespread protein degradation, clinically manifesting as sarcopenia or cachexia, and ultimately accelerating mortality. This narrative review examines the potential role of amino acids (AAs) in inhibiting tumor growth and counteracting protein–energy malnutrition—aiming to preserve muscle mass and nourish healthy cells while placing neoplastic cells in a state of metabolic stress. Methods: The analysis was conducted following the Standards for Reporting Qualitative Research guidelines. Results: Administration of targeted mixtures of essential amino acids (EAAs) has been shown to improve muscle mass, strength, and quality of life in patients with hypercatabolic conditions. Experimental in vitro and in vivo studies also suggest a potential inhibitory effect on tumor proliferation. However, increased availability of certain AAs may, in some cases, stimulate tumor growth, one reason why EAAs supplementation in cancer patients remains controversial. Conclusions: Despite prevailing concerns, emerging evidence indicates that supplementation with a complete, well-balanced EAAs formulation may be a valuable adjunct to standard cancer therapies. This approach could help correct cancer-associated protein imbalances, enhance patients’ quality of life, and create a metabolic environment unfavorable to tumor progression.

## 1. Introduction

Cancer is a multifactorial disease influenced by both environmental and genetic determinants. Environmental factors account for approximately 90–95% of cancer cases, whereas hereditary genetic factors represent a minor proportion. Within environmental determinants, dietary habits contribute to 30–35% of cases, and obesity to 10–20%. These two often interrelated factors are therefore responsible for roughly half of all tumor incidences [1].

Nutritional status plays a pivotal role not only in cancer prevention but also in prognosis. A substantial proportion of cancer patients (30–90%) present with malnutrition, resulting from many different causes as anorexia, reduced caloric intake, taste alterations, malabsorption, impaired digestion, metabolic disturbances, and insufficient food consumption. Moreover, virtually all cancer patients exhibit a hypercatabolic state, leading to systemic and tissue protein degradation. This protein catabolism results in metabolic derangements clinically manifested as sarcopenia, which, if untreated, progresses to cachexia, thereby accelerating mortality [2,3].

The risk of malnutrition is particularly high among older adults, with prevalence rates of 45% in community-dwelling individuals, over 50% in hospitalized patients, and 84–100% in residents of long-term care facilities [4]. These conditions further compromise clinical outcomes.

Clinical evidence demonstrates that administration of balanced mixtures of essential amino acids (EAAs), formulated according to human nutritional requirements, can prevent malnutrition, enhance muscle mass and strength, and improve quality of life in individuals with hypercatabolic conditions such as heart failure and age-related muscle loss [5,6,7,8]. Nevertheless, the role of amino acid (AA) metabolism in cancer remains complex and incompletely understood. Indeed, in certain contexts, increased availability of specific AAs may promote tumor growth. Consequently, the use of AAs supplementation in oncology requires careful evaluation.

This perspective narrative review aims to examine the effects and potential clinical utility of specific AA mixtures in both inhibiting tumor growth and counteracting protein–energy malnutrition and sarcopenia, with the goal of nourishing healthy tissues while placing neoplastic cells in a state of metabolic disadvantage, drawing on evidence from both experimental and clinical studies.

To improve the transparency of all aspects of this narrative and qualitative research, we followed the indications proposed by the Standards for Reporting Qualitative Research (SRQR) [9].

## 2. Cancer Cell Metabolism: An Overview

Only recently has it been understood that the metabolic plasticity of the tumor is fundamental to its development and spread. These mechanisms have been brilliantly analyzed in many reviews [10,11,12,13]. Therefore, in this paragraph, we will not delve into a detailed discussion but will instead provide a general overview.

It was Otto Warburg who first observed that tumor cells preferentially use aerobic glycolysis, even in the presence of oxygen, instead of oxidative phosphorylation [14,15]. The energy yield of the process is much lower than that of the Krebs cycle, but faster [15].

Subsequently, in tumor cells, it was shown that the tricarboxylic acid (TCA) cycle is often “remodulated” to produce biosynthetic molecules (such as fatty acids, glucose, nucleosides, and AAs) and to maintain redox balance [11,16]. AA metabolism is integrated with glycolysis and the TCA cycle to support tumor growth. Indeed, the diversion of 3-phosphoglycerate from glycolysis to serine synthesis provides 1C units for nucleotide synthesis; simultaneously, glycolysis maintains rapid ATP production [17]. Furthermore, glutamine supplies α-ketoglutarate (αKG) for TCA; this maintains pools of TCA intermediates that are exported as citrate (cytosolic acetyl-CoA for lipid synthesis) or oxaloacetate for transamination [18]. So, a distinguishing feature of tumor cells compared to healthy ones is the upregulation of glycolysis and alterations in the TCA cycle.

A common feature of all cancers is its growth by parasitizing the host’s energy and nutrients. It has been observed for a long time that tumors act as “nitrogen traps” after comparing the protein metabolism of normal tissue and of tumor tissue [19]. It was later observed that tumors utilized plasma proteins more efficiently than free [14C]-leucine. It was therefore concluded that tumor proliferation could be related to the ability of the tumor to trap plasma proteins and use the derived AAs “starving” the rest of the organism [20]. Subsequent studies have shown that plasma proteins, particularly albumin, are the main source of nutrition for the tumor from which they obtain AAs for their biosynthesis [21].

Today, it is established that the metabolism of cancer cells must be reprogrammed to maintain their survival and proliferation and may even hijack normal cells to create a tumor microenvironment (TME) for tumorigenesis and avoiding immune destruction [22].

In addition to metabolic reprogramming, tumor progression is influenced by multiple factors, including alterations in nutrient utilization, tissue of origin, cellular interactions, and the characteristics of TME [11,13]. Indeed, EAAs play a dual role in the TME. They are essential for immune cell function but are often co-opted by tumor cells to suppress antitumor immunity through competition, metabolism, and signaling [23,24,25,26]. Tumor cells and immunosuppressive cells (e.g., tumor-associated macrophages (TAMs) and myeloid-derived suppressor cells (MDSCs)) express enzymes like arginase 1 (Arg-1) and indoleamine 2,3 dioxygenase (IDO1), depleting arginine and tryptophan in the TME. This inhibits T and NK cell proliferation and function, while promoting regulatory T cell (Treg) expansion and exhaustion through upregulation of the protein named programmed death-1 (PD-1) via the aryl hydrocarbon receptor (AHR) pathway [24,27]. Therefore, modulating the EAAs metabolism could potentially be useful for promoting effective immune responses against tumor progression.

Due to the demands of cell growth and the need for newly synthesized molecules, protein biosynthesis is the most energy-demanding process that accounts for about 33% of total ATP consumption [28]. Therefore, mitochondrial activity and metabolism play a critical and fundamental role in promoting tumor proliferation, both by ensuring energy production and by providing essential metabolites for the synthesis of macromolecules and oncometabolites that characterize different types of tumors [29].

Metabolic reprogramming of tumor cells also involves lipids. Lipid desaturation increases the fluidity and flexibility of the cell membrane by facilitating the transport of membrane-associated proteins and lipids, promoting membrane remodeling, aiding cellular plasticity, survival, and migration [30,31,32]. Therefore, tumor cells increase lipogenesis and, to survive, require also a greater modification and high plasticity of lipid metabolism.

Finally, tumor cells require high availability of AAs, especially non-essential ones, to maintain intense activity [16,33,34,35,36]; therefore, they also reprogram AAs metabolism [13].

In parallel with the modulation of energy production via glycolysis, tumor cells also modulate numerous biochemical pathways to promote cancer development and spread. In particular, the mammalian/mechanistic target of rapamycin (mTOR) modulation plays an essential role. mTOR is a key cellular regulator that senses nutrients and energy to control growth, metabolism, and survival, with dysregulation linked to diseases like cancer and diabetes. Indeed, it has been identified as the main intracellular target for nutrition-induced cancer development [37,38]. mTOR is activated by various stimuli such as growth factors, stress, nutrient availability (especially nitrogen), glucose, ATP, and physical activity. In fact, mTOR is at the center of a complex network that controls protein synthesis, cell differentiation, growth, and proliferation [39]. Since the mTOR signaling pathway is closely related to tumor metabolism, this provides theoretical support for the combined application of mTOR inhibitors and drugs that interfere with tumor metabolism [38]. Other evidence indicates that mTOR activation may have a favorable role in cancer patients. In fact, exercise-induced mTOR activation has been found to be related to the prevention of some types of cancer [40,41]. Furthermore, mTOR activation can increase the organism’s tolerability to chemotherapy, inhibit autophagy, and improve prognosis [42]. mTOR functions mainly through two complexes named mTORC1 and mTORC2. mTORC1 promotes anabolic processes (protein/lipid synthesis) and inhibits catabolic processes (like autophagy) when nutrients and energy are abundant. mTORC2 regulates cell survival, cytoskeleton organization, and metabolism, especially through Akt (protein kinase B) signaling. Nutrients, especially some AAs, are key stimuli for the regulation and activation of the mTORC1 subunit. In fact, other stimuli are unable to effectively activate mTORC1 when there is a lack of AAs [43]. The main specific AAs needed to activate mTORC1 are leucine and glutamine [44]. Therefore, AAs can modulate cell metabolism and control cell fate by regulating cell survival and death, and the molecular mechanism is mainly mediated by mTORC1 [45].

In summary, the altered metabolism of tumor cells is not only a key driver of their proliferation, but also a potential Achilles’ heel that can be targeted to counteract them. Emerging evidence indicates that dietary interventions restricting entire nutrient classes—such as fasting or the limitation of glucose and selected AAs—can confer anticancer benefits by suppressing tumor progression [46].

## 3. The Multiple Roles of Amino Acids in Cancer Cells

Like any other cells, tumor cells require all AAs to build proteins and proliferate. As previously described, AAs have different and important roles in control of the TME, which is crucial for tumor cell metabolism; therefore, they could be a promising target to inhibit their development and spread [47].

A key question to consider is as follows: do AAs always activate mTORC1, stimulating cell growth and proliferation, favoring anabolism, regardless of their type or molar ratio?

As is known, AAs can be classified as non-essential (NEAAs) or essential (EAAs), depending on their potential to be synthesized endogenously or not [48]. In cancer cells, alterations in AAs metabolism contribute to their metabolic rearrangement and provide the building blocks and energy necessary for proliferation and spread. Historical studies have identified that the main source of AAs used by cancer in tissues that act as reservoirs during prolonged fasting are skeletal muscles and skin [49]. It has been known for half a century that 12 AAs are essential for the survival of tumor cells in vitro culture [50]. Notably, cancer cells are particularly avid for the NEAAs as glutamine, serine, proline, and glycine, which are most abundant in food proteins [51,52]. These NEAAs are fundamental for metabolic processes of tumor cells as the synthesis of pyrimidines and membrane lipids, as well as for integrating the Krebs cycle intermediates necessary not only to produce energy but also fundamental to support global cells’ metabolism [21].

### 3.1. Glutamine

Glutamine is a rate-limiting molecule in the cellular reproductive cycle. Indeed, data show that tumor cells generally arrest S phase when deprived of glutamine [53]. ASCT2(SLC1A5) is the main glutamine transporter in many cancer cell lines. It is regulated by multiple cancer-associated transcription factors. Therefore, since ASCT2 transports glutamine for cancer cells’ consumption, its inhibition exerts an antitumor action by promoting apoptosis [16]. In addition, glutamine also plays a crucial role both for the biosynthesis of proteins, nucleotides, and antioxidant molecules (glutathione), and for energy production in the tricarboxylic acid (TCA) cycle as a precursor of αKG [16,54,55,56]. Aerobic oxidation of glutamine provided more than half of the cell energy requirements in HeLa cells and, after substitution of glucose, about 98% of the energy was derived from glutamine [56]. So, glutamine is used as a major source of cell energy, rather than for incorporation into proteins.

Glutamine is metabolized through glutaminolysis, a process catalyzed by glutaminase 1(GLS1) or GLS2 to produce glutamate, and activates mTORC1, regulated by the AMP-activated protein kinase (AMPK)-mTORC1 signaling axis [16]. AMPK, a highly conserved serine/threonine kinase complex composed of α, β, and γ subunits, acts as a crucial “fuel gauge” in eukaryotic cells. It becomes activated when cellular energy is low, signaled by increased AMP/ATP or ADP/ATP ratios, such as during hypoxia, glucose deprivation, or intense muscle activity [57].

The activation of mTORC1 by glutaminolysis during nutritional imbalance led to the anomalous inhibition of autophagy and a subsequent form of apoptosis named glutamminoptosis [58]. AMPK activation blocks both glutamine-dependent mTORC1 activation and glutaminoptosis in vitro and in vivo. Furthermore, glutamine is used for asparagine synthesis and GABA shunting to produce ATP and inhibit AMPK, independently of glutaminolysis [59]. Asparagine has been identified as an exchange AA factor that regulates mTORC1, nucleotide biosynthesis, and proliferation [60]. So, glutamine is sufficient to sustain the production of ATP in the absence of any other AAs, following a glutaminolysis-independent mechanism. The increased uptake and metabolism of GABA in breast to brain metastatic cells was linked to an increase in NADH levels in the microenvironment, conferring a proliferative advantage to the tumor [61]. Glutamine metabolism promotes mTORC1 activation through two distinct but parallel pathways: a primary αKG-dependent pathway and a secondary ATP/AMPK-dependent pathway. These pathways regulate the mTORC1-macroautophagy/autophagy pathway [62]. The metabolic connection between glutamine and mTORC1 could therefore represent a key point for preventing the growth of tumor cells.

### 3.2. Leucine

Leucine also acts as an allosteric activator of glutamate dehydrogenase, which is consequently necessary for glutaminolysis induction [63]. The addition of glutamine and leucine at similar concentrations in a complete culture medium to AA-starved cells promoted the activation of apoptotic cancer cell death through glutaminolysis-induced activation of mTORC1 via inhibiting autophagy. This highlights that under such conditions, inhibition of mTORC1 results in the survival of cancer cells [58]. In addition, previous reports showed that increasing intracellular αKG levels also induces cancer cell apoptosis in vivo [64].

As previously described, short-term glutaminolysis activates mTORC1. In contrast, long-term activation of glutaminolysis, or its end-product αKG, maintains mTORC1 activity for at least 72 h, favoring the activation of apoptosis [59]. In AA deficiency, mTORC1 inhibition promoted cell survival. Considering that mTORC1 is hyperactivated in approximately 80% of solid tumors [65], and that the TME is frequently nutrient-deprived, sustained mTORC1 activity under nutrient-limited conditions imposes substantial metabolic stress on tumor cells [66]. Indeed, glutaminolysis-mediated activation of mTORC1 inhibited autophagy, a process crucial for the survival of the cells upon nutrient deprivation. In contrast, mTORC1 inhibition facilitates epithelial–mesenchymal transition, thereby enhancing cancer cell migration [67].

Leucine has opposing effects on tumor cells, acting both as a promoter and inhibitor of proliferation. It promotes tumor growth by activating the mTORC1 signaling pathway, which enhances protein synthesis, lipogenesis, and cell proliferation in cancers such as hepatocellular carcinoma, breast, pancreatic, and colorectal cancers [36,68]. It has been proven that 5% leucine supplementation in mouse pancreatic cancer models increased tumor growth [69]. In addition, in bone sarcomas, leucine metabolism supports energy metabolism and tumor survival [70].

**Figure 1:**
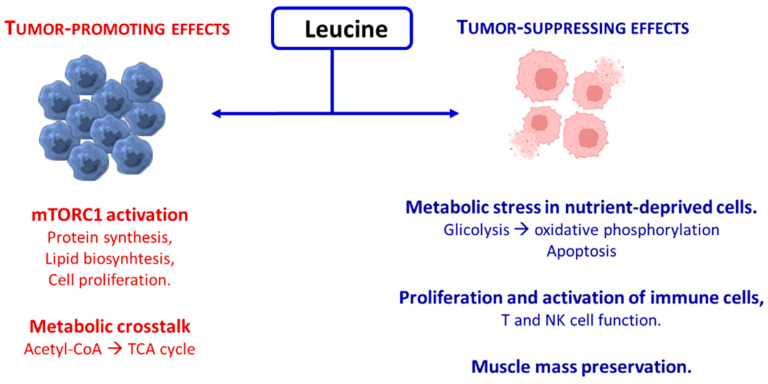
Schematic representation of the dual effects of leucine on tumor progression.

On the contrary, leucine supplementation suppresses tumor growth or supports antitumor responses, inducing tumor cell apoptosis and oxidative stress by shifting metabolism toward oxidative phosphorylation. Furthermore, it inhibits the IGF-1/PI3K/Akt/mTORC1 axis and increases levels of the tumor-suppressor p53. In addition, it enhances antitumor immunity by inhibiting regulatory T cells and reducing immune checkpoints like PD-1; it can also compete with kynurenine in the tumor microenvironment, supporting T cell and NK cell function [16]. In hepatocellular carcinoma cell lines, combining leucine supplementation with epigenetic therapy (e.g., histone deacetylase inhibitors) led to anti-proliferative effects, decreasing IGF-1 and p70 S6K, while increasing p53 and caspase-3 activity [71]. In summary, anti- or pro-tumorigenic effects of leucine supplementation in various cancers should be further investigated to achieve clear conclusions. The dual effects of leucine on tumor progression are schematized in Figure 1.

### 3.3. Serine

Serine is the second frequently used AA fundamental for cancer cell survival. Serine has been shown to promote the growth of a variety of tumors through the lipid metabolism pathway (sphingolipids, phospholipids, and ceramide) [72,73], AAs homeostasis, epigenetic regulation, and redox balance [74,75,76]. Moreover, cancer cells exploit serine as a major source of one-carbon units to sustain the folate cycle, thereby rendering them dependent on either extracellular serine uptake or de novo serine synthesis to support maximal growth and proliferation [75,77]. Serine biosynthesis is activated by transcription factors or other mediators in rapidly growing tumors [78]. ATF3 and ATF4 are the major transcription factors that directly bind to the serine biosynthesis gene promoter to activate serine synthesis and growth of different tumors [79]. 3-Phospho-glycerate, an intermediate of glycolysis, is the main substrate for the synthesis of serine, which consists of a series of biochemical reactions catalyzed by four cytoplasmic and one mitochondrial enzyme [12]. Both synthesized and ingested serine are known to functionally support cancer development at all stages of tumorigenesis, from initiation to metastasis [12]. For these reasons, intervening on the serine biosynthesis pathway, including dietary restriction, seems a promising and relevant target to improve the effects of chemotherapy in cancer patients. In fact, serine restriction, both endogenous and exogenous, reduces tumor resistance to 5-FU or other chemotherapeutic treatments [12]. However, a recent in vivo study on murine colon cancer (CT26 cell line) with animals fed a special diet rich in all EAAs and containing serine, showed significant cancer cell death in vitro and a substantial slowdown of tumor progression in vivo [16].

### 3.4. Arginine

Arginine is a NEAA considered semi-essential that plays a key role in cellular metabolism, synthesis of protein, nitric oxide, and polyamines, and immune system regulation. In addition, it is a direct activator of mTOR, a nutrient-sensing kinase strongly implicated in carcinogenesis. Some tumors exhibit a deficiency or absence of key enzymes for endogenous arginine synthesis (e.g., arginosuccinate synthase or ASS1). In over 70% of tumors, ASS1 transcription is suppressed, making them dependent on external arginine supplies for proliferation [80]. This has led to the development of therapies that deprive tumors of arginine, blocking their growth [80,81]. Furthermore, arginine is essential for T-lymphocyte function and the production of nitric oxide (NO), a mediator with variable effects in the tumor microenvironment [82]. However, some tumors express arginase, an enzyme that degrades arginine, contributing to local immunosuppression and thus promoting tumor progression [82]. The use of enzymes such as arginase or arginine deaminase to reduce arginine availability in the TME is the subject of clinical trials for the treatment of various types of cancer [83].

### 3.5. Asparagine

Asparagine is an NEAA widely used by cells for the production of other nutrients such as glucose, proteins, lipids, and nucleotides. Asparagine can be produced by the cell from aspartate and glutamine by the enzyme asparagine synthase and plays a crucial role in the growth and spread of certain types of cancer [84]. Some tumors, particularly acute lymphoblastic leukemia (ALL) cells and solid tumors such as breast cancer, have low or absent expression of asparagine synthase and are therefore dependent on asparagine circulating in the blood for protein synthesis, proliferation, and metastasis [85,86]. The enzyme asparaginase is used to degrade asparagine in the bloodstream, reducing its availability and hindering tumor growth, especially in ALL [87]. However, in solid tumors, asparagine restriction alone does not provide therapeutic efficacy. In fact, tumor cells initiate reprogramming processes in response to asparagine deprivation [88].

### 3.6. Proline and Glycine

The other two NEAAs needed by tumor cells are proline and glycine. They are very abundant in collagen; therefore, the dermis in primis represents an excellent endogenous reservoir. It has been demonstrated that proline availability influences collagen synthesis, promoting the maturation, plasticity, and heterogeneity of cancer cells [89]. Glycine is biosynthetically linked to serine, and both are important precursors for the synthesis of proteins, nucleic acids, and lipids, which are essential for the proliferation of tumor cells. In addition, their biosynthesis influences the antioxidant capacity of cells, promoting tumor homeostasis [90]. Moreover, studies on the proliferation rates of various tumor cell lines have revealed that glycine is consumed by cells in a highly proliferative state, while it is released by slowly proliferating cells [91].

### 3.7. Essential Amino Acids (EAAs)

While tumor cells predominantly rely on NEAAs in large quantities [10], experimental evidence demonstrates that EAAs are also indispensable for sustaining tumor cell survival in culture [52,53,54,73]. It is well established that cancer cells increase the uptake of the branched-chained AAs (BCAAs), mostly leucine, to support their rapid proliferation through mTORC1 activation [92,93], while the other EAAs are necessary for protein synthesis and metabolism support of neoplastic cells.

To fulfill their metabolic requirements, cancer cells exploit macropinocytosis to internalize extracellular proteins, preferentially albumin, as a nutrient source, subsequently catabolizing them to release AAs, with a pronounced enrichment in NEAAs, for which they exhibit elevated uptake and utilization [21]. Beyond albumin, cancer cells utilize AAs derived from the breakdown of host tissues, notably skeletal muscle and skin, leading to sarcopenia, cachexia, and hypoalbuminemia, clinical indicators of disrupted systemic protein homeostasis and poor patient outcomes [49].

Considering what has been reported above, it is important to underline that limiting and/or modulating AAs availability in the TME and consequently inside the cancer cells could be an effective strategy to influence the tumor growth [94]. However, it is important to emphasize that reducing the availability of a single AA or group of AAs (as BCAAs) not only affects tumor cells, but also healthy cells that will have difficulty synthesizing proteins that ensure normal metabolism and the function of organic systems. Paradoxically, in the process of targeting the tumor, the host organism becomes progressively debilitated, resulting in widespread disruption of protein homeostasis. In hypercatabolic states such as cancer, preserving metabolic integrity and promoting an anabolic state, including muscle mass restoration, demands sufficient energy intake and a balanced supply of AAs, particularly all EAAs [6]. Since NEAAs are more represented than EAAs in dietary proteins, meeting the needs of EAAs is certainly much more difficult than meeting those of NEAAs [52]. Indeed, modulating the intake of some single AAs may be useful in experimental models [95], but it is far from practical application in humans. In conclusion, the intake of nitrogen from AAs in both health and tumor cells is extremely complicated and multivarious must always be considered in its complexity [96].

## 4. Amino Acids in Experimental Models of Cancer

### 4.1. The Traditional Approach: A Single AA as a Drug

Despite the recognized importance of NEAAs in cancer cell metabolism, their therapeutic restriction has shown limited efficacy, as tumor cells can synthesize NEAAs de novo from metabolic intermediates and/or EAAs as needed [97]. Indeed, as already mentioned, cancer cells can increase BCAAs metabolism to stimulate tumor growth [91,92].

Some studies have found that leucine supplementation promotes cancer growth and aggressiveness [36] and, although it was observed as early as 1954, that tumors utilize plasma proteins (above all albumin) more efficiently than free [14C]-leucine [20]. Leucine deprivation inhibited cell proliferation and induced apoptosis of MDA-MB-231 and MCF-7 breast cancer cells in fatty acid synthase (FASN)-dependent manners [98]. These observations suggest that the availability of leucine may have pro-tumorigenic effects and antitumor effects [36]. However, other studies show that a diet rich in leucine (3%) led to a metabolic shift in the Walker-256 rat tumor towards a less glycolytic profile associated with a decrease in tumor aggressiveness and the number of metastatic sites [99]. To date, evidence remains insufficient to establish a definitive causal relationship between leucine and cancer cell proliferation [34].

Other studies showed that a lower-BCAAs diet impedes pancreatic ductal adenocarcinoma (PDAC) development in mouse models. Indeed, BCAA-aminotransferase-2 (BCAT2)-mediated BCAAs catabolism is critical for the development of PDAC [100]. Furthermore, high levels of dietary BCAAs intake promoted colon-rectal cancer (CRC) tumorigenesis in chemical-induced CRC and xenograft mouse models through inhibition of BCAAs metabolism and chronic activation of mTORC1 [101]. Both the mRNA and protein levels of BCAT2 were decreased in tumor tissues of patients with CRC compared to those in normal tissues. Furthermore, the accumulation of BCAAs caused by BCAT2 deficiency facilitated the chronic activation of mTORC1, thereby mediating the oncogenic effect of BCAA [101].

In addition, methionine-deficient diets but supplemented with cysteine, taurine, or both have demonstrated marked antitumor activity in animal models of colon cancer (CT26 cell line), as well as increased survival of mice with disseminated ovarian cancer (ID8 Tp53−/− cells) and renal cell carcinoma [102]. This is not surprising because methionine occupies the N terminus of all eukaryotic proteins, as it corresponds to the start codon of mRNA for protein synthesis. Therefore, its deficiency hinders the synthesis of new proteins (structural and enzymatic) that are essential for the tumor cell to multiply.

Unfortunately, the findings from previous studies on the role of AAs in inhibiting or promoting tumor progression have been inconsistent and often contradictory. There is no clear evidence that a single AA, or a small group of AAs, can independently influence tumor growth. This may be because such studies have tended to treat individual AAs (e.g., leucine) or small combinations as conventional drugs, assuming they exert specific, isolated pharmacological effects. In reality, AAs function as integral components of a highly interconnected metabolic network, engaging in numerous interdependent biochemical pathways. Modifying the concentration of one or a few AAs can markedly disrupt the balance between EAAs and NEAAs (EAA/NEAA ratio), thereby influencing cellular metabolism in complex and often unpredictable ways, sometimes producing effects opposite to those intended [103,104,105].

Before proceeding further, it is essential to address a critical concept: the role of AAs within the broader metabolic framework of living organisms.

Unlike conventional pharmacological agents, a single AA should not be viewed in isolation, but rather as a metabolically active molecule that interacts with and modulates numerous components within an intricate biochemical network. Pharmacology often focuses on single drug entities interacting with specific receptors to elicit a therapeutic effect. In fact, drugs exhibit high affinity and selectivity for specific molecular targets, often cell surface receptors, to trigger downstream signaling cascades and achieve a desired therapeutic outcome [106]. However, this reductionist approach may not be entirely applicable to endogenous molecules like AAs. These molecules are not xenobiotics but rather integral components of the overly complex and interconnected network of metabolism, participating in a multitude of biochemical reactions essential for life. AAs occupies central roles within the metabolic web, serving as substrates for protein synthesis, precursors for various biomolecules, energy sources through catabolic pathways, and key regulators of metabolic enzymes and signaling pathways [107,108].

We believe that to achieve specific and predictable metabolic effects using AAs, a strategy that leverages the synergistic interactions within the metabolic network is essential. Because metabolic pathways are interdependent and modulating one point in a pathway can have consequences elsewhere in the network, we postulate that combinations of AAs can be used to exert coordinated effects at multiple regulatory points within a specific pathway or across interconnected pathways to achieve a desired outcome. In addition, specific combinations of AAs can exhibit synergistic effects, where the combined effect is greater than the sum of the individual effects. This can lead to more potent and targeted modulation of metabolic pathways. Therefore, the research approaches should shift towards understanding and exploiting the synergistic actions of carefully designed and balanced EAAs mixtures, because they can provide a more physiological and sustainable approach to metabolic modulation. For these reasons, we believe that the conflicting results of previous studies depend on seeking and considering the effect of a single AA, when instead the proteinogenic AAs are twenty and their stoichiometric ratios are in dynamic equilibrium as a function of cellular metabolism. Crucially, the rate-limiting factor in this system is the simultaneous availability of all EAAs, which governs the efficiency and balance of anabolic processes [50]. For instance, the anabolic and metabolic effects of leucine are contingent upon the availability of all other EAAs in sufficient amounts to support and sustain cellular biosynthesis. Nevertheless, a substantial body of research has investigated the impact of individual AAs on cellular metabolism in isolation, disregarding these interdependencies [109]. Only a limited number of investigations have attempted to distinguish the metabolic impacts of EAAs from those of NEAAs [110]. This distinction is even less explored in the context of tumor cells, despite evidence showing that alterations in the EAA/NEAA ratio can significantly influence cellular metabolism [103,104]. Moreover, the restriction of specific EAAs and NEAAs, including leucine, with or without valine and isoleucine, as well as glycine, serine, glutamine, asparagine, and methionine, has been shown to affect tumor growth [73].

### 4.2. The Innovative Approach: A Specifically Designed EAAs Mixture

It is well established that in all cells, protein synthesis and autophagy are dynamically interconnected and tightly regulated processes. They are influenced both by the availability of substrates, particularly AAs, required for biosynthesis, and by fluctuations in cellular energy status (ATP levels), which reciprocally affect one another. Among intracellular processes, protein synthesis is one of the most energy-intensive, consuming substantial amounts of ATP and converting it into AMP during the formation of hundreds of peptide bonds required to assemble a single protein. As ATP consumption increases, AMP levels rise proportionally. These shifts in ATP and AMP concentrations directly modulate the activity of key regulatory pathways—specifically mTORC1 and AMPK—that determine the timing, frequency, and extent of both protein synthesis and autophagy [34]. Based on this understanding, it can be inferred that altering the EAA/NEAA ratio in the TME to favor EAAs may stimulate robust protein synthesis, leading to elevated ATP consumption and a subsequent increase in ADP and AMP levels [103]. The induction of protein synthesis promoted by excess EAAs could be inhibited by the reduced availability of NEAAs needed to complete the synthesis. As a result, the cell must synthesize NEAAs endogenously, further increasing ATP consumption and thereby promoting autophagy. However, autophagy can also be triggered by mTORC2 independent of mTORC1 [111]. These authors demonstrated that fasting reduces the availability of NEAAs, particularly glutamine, triggering autophagy independently of TORC1 inactivation. When glutamine demand is not met, especially due to insufficient intake of EAAs precursors, the cell resorts to muscle protein catabolism, contributing to cancer-related sarcopenia and cachexia [111].

**Figure 2:**
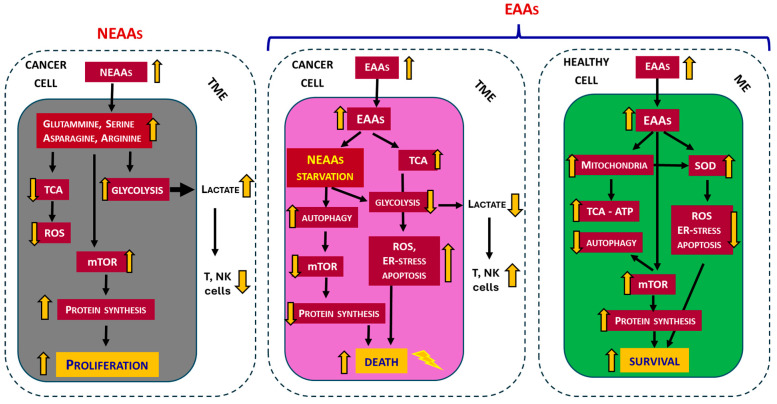
Schematic summary of opposing outcomes driven by excess non-essential (NEAAs) versus essential amino acids (EAAs) in cancer and healthy cells. NEAAs promote tumor progression through metabolic support and tumor microenvironment (TME, delimited by the dotted line) remodeling, whereas EAAs induce selective cancer cell death while preserving and enhancing normal cell viability and function. ME = microenvironment. ER = endoplasmic reticulum. TCA = tricarboxylic acid cycle. ATP = Adenosine triphosphate. ROS = Reactive oxygen species. SOD = Super oxide dismutase. mTOR = mammalian target of rapamycin. Immune cells: T = T-lymphocytes, NK = Natural-killer-lymphocytes. Arrow yellow: up = increase, down = decrease.

Notably, it was observed that the addition of a mixture containing all EAAs in stoichiometric ratio to the culture medium of in vitro isolated three human tumor cell lines [melanoma (M14), breast cancer cell (MCF7) and colon cancer cells (HCT116)] caused the death of the tumor cells [112] and, at the same time, enhanced the effect of the chemotherapeutic drug Doxorubicin [113]. Subsequently, in five human tumor cell lines, it has been demonstrated that altering the EAA/NEAA ratio with excess of EAAs (84%), it is possible to inhibit proteasomes and promote autophagy and triggering apoptosis without damage to normal cells [33]. Recently, in vivo experiments showed that an EAAs-rich diet reduced tumor growth, induced endoplasmic reticulum (ER) stress, and inhibited mTOR activity [34]. In tumor cells in vitro, the EAAs mixture increased BCAAs oxidation, decreased glycolysis, ATP levels, redox potential, and intracellular NEAAs content. EAAs-induced NEAAs deficiency led to increased ER stress, mTOR inactivation, and apoptosis, without affecting the survival and proliferation of non-tumor cells [34]. Moreover, it has been demonstrated that in vivo the administration of a specific mixture of all EAAs in a stoichiometric ratio increases intracellular EAAs levels while reducing concentrations of glutamate, glycine, aspartate, and alanine. This biochemical profile mimics a starvation-like response, primarily due to glutamate depletion, which subsequently activates mitochondrial catabolism of BCAAs and inhibits glycolysis (the Warburg effect) in favor of TCA cycle activation. These metabolic shifts induced by excess of EAAs lead to tumor growth suppression, increased ER stress, reduced mTORC1 activity, enhanced apoptosis, and tumor cell death. In contrast, the metabolic and biosynthetic functions of non-tumor cells are effectively supported by the availability of EAAs [34] (Figure 2). It is therefore evident that in tumor cells the “classical” concept of EAAs and NEAAs does not follow the traditional definition, as EAAs and NEAAs play highly flexible roles in protein synthesis and energy in tumor cells [16].

More recent in vitro and in vivo evidence, including studies on murine (CT26) and human (HCT116) colon cancer cells, indicates that an excess of EAAs in the culture medium or diet exerts significant cytostatic effects on cancer cells when provided at levels sufficient to markedly increase the EAA/NEAA ratio, typically <<0.9 in dietary proteins, to value well >>1. Furthermore, an additional noteworthy finding is that animals bearing tumors and fed an EAAs-enriched diet exhibit preservation of muscle mass [35]. The EAAs-rich mixture used in these studies is summarized in Table 1. It is important to emphasize that the EAAs mixture described in Table 1 has proven effective in preventing and treating hypercatabolic conditions in both experimental models and clinical settings [6,7,8]. This efficacy is attributed to its ability to enhance cellular metabolism by stimulating mitochondrial biogenesis and activity, activating mTORC1, and inducing eNOS expression as previously described [114].

**Figure 3:**
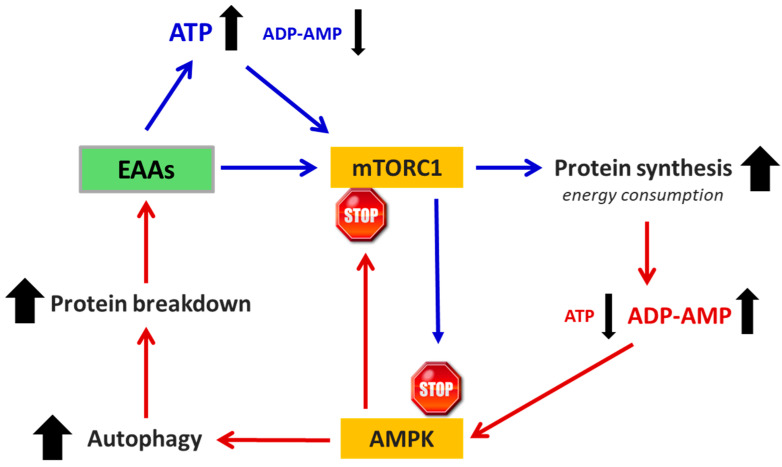
Schematic representation of the link between EAAs availability, ATP production, and mTOR activation. ATP, ADP, AMP = adenosine tri-, di-, and monophosphate. AMPK = AMP-activated protein kinase. mTORC1 = mammalian target of rapamycin subunit 1. The blue arrows indicated the anabolic pathway, the red ones the catabolic pathway. Black arrow up = increase. Black arrow down = decrease.

If the EAA/NEAA ratio in the tumor cell is shifted in favor of EAAs, it favors the consumption of substantial amounts of ATP to build the thousands of peptide bonds needed for protein synthesis, consequently leading to an increase in ADP and AMP. This activates AMPK, which, in turn, inhibits mTORC1 and activates autophagy, providing substrates to support the new production of ATP and a decrease in the availability of NEAAs (Figure 3). These changes create a vicious cycle that inhibits cell proliferation and induces apoptosis [107].

**Table 1:** Composition of EAAs-mix for in vitro and in vivo experiments. BCAA = branched-chain AAs. The EAAs mix provided nitrogen as free AAs, where the EAAs are in excess (84%) compared to NEAAs (16%) (EAA/NEAA = 6.14). L-cystine (NEAA) was added to satisfy the requirements for sulfur AAs while keeping methionine content to a minimum. Additionally, L-serine (NEAA) was included to optimally sustain the folate and methionine cycle, as well as energy production. Ornithine alpha-ketoglutarate is a key intermediate in the Krebs cycle and serves as a precursor for polyamines, proline, and glutamine. *N*-acetylcysteine is a derivative of cysteine that acts mainly as a precursor to glutathione, a major intracellular antioxidant.

Free AAs Composition of EAAs-Mix	%
L-Leucine (BCAA)	13.53
L-Isoleucine (BCAA)	9.65
L-Valine (BCAA)	9.65
L-Lysine	11.6
L-Threonine	8.7
L-Histidine	11.6
L-Phenylalanine	7.73
L-Methionine	4.35
L-Tyrosine	5.80
L-Tryptophan	3.38
L-Cystine/Cysteine	8.20
L-Serine	2.42
Ornithine-αKG	2.42
*N*-acetylcysteine	0.97

Globally, these studies indicate that an excess of EAAs, leading to an increased EAA/NEAA ratio, exhibits remarkable antiproliferative properties, negatively influencing cancer cell survival, and supporting the health of normal cells (Figure 2).

## 5. Amino Acids in Cancer Patients

In cancer therapy, AA metabolism can be approached from different angles: inhibition of either AA transporters, AA biosynthesis, or restriction/depletion of a single AA. For example, pharmacological inhibition of glutaminase has yielded encouraging results by inhibiting tumor cell proliferation in several cancer types, including triple-negative breast cancer, acute myeloid leukemia, and non-small cell lung cancer [94]. Other evidence suggests that the administration of the enzyme asparaginase in the treatment of pediatric acute lymphoblastic leukemia improved the cure rate [115]. Indeed, while most cells express asparagine synthase, the enzyme that converts aspartate to asparagine, in immature and leukemic lymphocytes, its expression is insufficient, making these cells auxotrophic for asparagine. Consequently, treatment with asparaginase kills leukemic cells, although it is associated with several manageable acute toxicities [116].

In the clinical setting, when tumor cells selectively depend on the exogenous supply of specific AAs, their depletion in the blood flow will lead to intracellular deficiency of the same, cessation of synthesis, hence of proliferation, and, finally, induction of apoptosis [117]. Recently, a clinical study enrolled six healthy, middle-aged participants who were placed on a low-methionine diet (~2.92 mg/kg/day) for three weeks, representing an approximately 83% reduction in daily methionine intake. In this regimen, 75% of total protein was supplied by a methionine-free medical formula (Hominex-2, Abbott Nutrition, Columbus, OH, USA), while the remaining 25% was derived from low-methionine foods such as fruits, vegetables, and refined grains. Methionine restriction has been shown to cause metabolic changes like those observed in colorectal tumor-bearing mice, including inhibition of nucleotide synthesis metabolism [118]. It is therefore hypothesized that methionine restriction in cancer patients could inhibit tumor progression similarly to what is well documented in experimental mouse models [119]. Currently, AAs under investigation in clinical trials (phase I or II) for therapeutic deprivation include asparagine, glutamine, arginine, and methionine [94].

AAs restriction strategies may hold theoretical promise for human cancer therapy, partly due to their lower toxicity compared to conventional chemotherapeutics. However, prior to clinical translation, a more comprehensive understanding of the metabolic dependencies and TME specific to each cancer type is essential. Furthermore, the therapeutic efficacy of interventions that modulate AA availability is influenced not only by tumor type but also by the anatomical context and functional status of the immune system [119]. It is also important to recognize, as previously noted, that AA restriction impacts not only malignant cells but also normal tissues. Consequently, a detailed characterization of its effects on healthy cells is essential. Overall, therapeutic approaches based on the selective restriction of specific AAs remain insufficiently characterized and warrant further investigation [120].

Another approach involves integration with BCAAs. In a multicenter, randomized, controlled trial, long-term oral supplementation with BCAAs (12 g/day) in cirrhotic patients has been shown to be effective in limiting the development of liver cancer [121,122]. Higher levels of dietary BCAAs intake are not associated with an increase in CRC risk, but they may be related to a reduced risk of sigmoid colon cancer [123]. Conversely, high blood levels of BCAAs are an early event in the development of pancreatic cancer [124]. Furthermore, evidence indicates that elevated levels of BCAAs in plasma are positively associated with pancreatic cancer risk [125]. However, epidemiological studies have shown that the incidence of liver cancer in cirrhotic patients is reduced by chronic intake of BCAAs [126,127,128]. The existing literature is fragmented and frequently contradictory, largely due to heterogeneity in experimental designs and the specific AAs examined. In our view, a major limitation lies in the predominant focus on branched-chain AAs (BCAAs) in isolation, rather than on the use of a complete mixture of all EAAs in appropriate stoichiometric ratios.

### Amino Acids as Prevention or Treatment of Cancer Cachexia

Cancer patients, especially those experiencing cachexia, inflammation, or undergoing intensive treatments (chemo- or radiotherapy) require higher protein intake than healthy individuals. Guidelines recommend a total protein intake ranging between 1.0 and 2.0 g/kg/day, with 1.2–1.5 g/kg/day often cited for intermediate needs and up to 2.0 g/kg/day in more severe cases. Correspondingly, EAAs targets are suggested to be ≥1.2 g/kg/day in hypercatabolic or cachectic patients [129].

Most research on AAs in cancer patients has focused on strategies to prevent or attenuate the loss of muscle mass (MM). Cancer-associated malnutrition is highly prevalent and negatively affects both clinical outcomes and quality of life. In this population, the most pronounced manifestation of malnutrition is a marked reduction in MM, ranging from sarcopenia to cachexia, which may occur independently of body weight or fat mass [130]. The reported prevalence of malnutrition varies widely, from 20% to 70%, depending on tumor type, disease stage, and clinical context [131,132]. Systemic inflammation and other metabolic disturbances are present in 34% of patients with colorectal cancer (CRC) undergoing curative treatment, in 75% of cancer patients at hospital admission [133], and in 55–85% of patients with advanced disease [134].

Cancer cachexia is characterized by a negative energy balance and profound disturbances in protein metabolism. One major contributor to muscle loss is the increased splanchnic sequestration of AAs, particularly EAAs, to support hepatic activity in response to the intense inflammatory state [135]. As the primary reservoirs of AAs, skeletal muscles are significantly depleted to meet the elevated systemic demand for EAAs, including branched-chain amino acids (BCAAs). Additionally, the heightened energy requirements may stimulate muscle protein breakdown to supply AAs substrates for gluconeogenesis and glycogen synthesis, further exacerbating the loss of skeletal muscle mass [136]. Consequently, the protein requirements of cancer patients are typically higher (1.2–2.0 g/kg/day) than those of healthy individuals (0.8 g/kg/day), especially in the presence of additional catabolic factors such as physical inactivity or age-related comorbidities [131,132].

Treatment strategies and clinical outcomes for malnutrition in cancer patients differ according to the presence or absence of underlying metabolic derangements. “Starvation-type” malnutrition results primarily from a straightforward deficit in nutrient and energy intake, whereas “inflammatory-type” malnutrition, commonly termed cancer cachexia, is driven by complex metabolic alterations associated with systemic inflammation [132]. The anabolic resistance observed in these patients can be mitigated through the administration of AA mixtures enriched with leucine. Among the essential amino acids, BCAAs have been the most extensively studied in oncologic nutrition due to their potent ability to stimulate muscle protein synthesis. Unfortunately, the studies have many critical issues due to different causes that make the results heterogeneous and contrasting [137]. However, as described in a previous section (Section 3.2), a review of the literature suggests that leucine enhances protein synthesis through activation of mTORC1, while simultaneously reducing protein degradation. This dual action contributes to the preservation of MM, augments the beneficial effects of physical activity on sarcopenia, and may also attenuate cancer progression and prevent cancer-associated cachexia (see Figure 1) [138,139]. In patients with hypercatabolic syndrome, as well as in experimental models, administration of a mixture of all EAAs, formulated in a human-tailored stoichiometric ratio, has been shown to counteract sarcopenia and protein dysregulation. This intervention promotes anabolism by enhancing mitochondrial activity, inducing mitochondrial biogenesis, and activating both mTORC1 and endothelial nitric oxide synthase (eNOS) [6,140,141,142]. Collectively, these effects not only support muscle protein synthesis but also improve overall cellular energy metabolism, highlighting the clinical importance of targeted nutritional support in enhancing physical performance, particularly in hypercatabolic or malnourished patients [143].

## 6. Does Food Integration with EAAs Promote Cancer?

It is commonly agreed that nutritional choices can influence the risk of developing certain cancers [144]. Major organizations, including the National Cancer Institute, Cancer Research UK, and the European Society for Clinical Nutrition and Metabolism (ESPEN), have issued nutritional guidelines for cancer patients that recommend a “healthy” diet, with adequate energy and nutritional intake, particularly regarding the protein amount [131]. Unfortunately, nothing is said about the quality of the proteins. Many nutritional approaches have investigated fasting and caloric restriction, fat and carbohydrate balance, the ketogenic diet, protein restriction, and modulation of EAAs or NEAAs. Since cancer cells are highly dependent on NEAAs, research has focused primarily on their dietary manipulation [120]. However, it should be emphasized that dietary manipulation affects not only the tumor but also all physiological systems. Under normal conditions, approximately 70% of the EAAs released through muscle protein breakdown are reincorporated into newly synthesized muscle proteins. In patients with neoplastic disease, this recycling efficiency is markedly reduced; instead, amino acids are released into circulation and subsequently deaminated to provide energy and sustain other metabolic processes [6,105]. As a result, supplementation with BCAAs alone, despite their elevated requirement, is insufficient to sustain muscle protein synthesis. This is primarily because the limited availability of other EAAs rapidly becomes the rate-limiting factor [105]. Therefore, an anabolic state cannot occur without the availability of all EAAs in adequate quantities.

The positive effect of EAAs supplementation has been observed in numerous experimental conditions, including chemotherapy [113], and is based on the stimulation of anabolism by activating the enzyme eNOS with consequent mitochondrial genesis and reduction in reactive oxygen species (ROS) [142], as well as the activation of mTORC1, with increased protein synthesis [145]. To meet its demand, tumors extract nutrients from the surrounding TME and also manipulate the behavior of stromal cells. In addition, autophagy, both in tumor and stromal cells, helps to maintain nutrient availability during tumor development [120]. Within the cell, a balanced protein synthesis and degradation is crucial for preserving cell homeostasis. Hence, increased proteolysis and reduced protein synthesis (protein’s disarrangement) have been associated with the severe depletion of body protein reserves, eventually resulting in malnutrition. This may impact the progression of disease-associated conditions, including tumors [128].

In recent years, the potential of leucine to improve muscle wasting in cancer cachexia has been extensively studied [36]. However, it should be noted that in these studies, patients supplemented with leucine obviously received a protein-rich diet from which they obtained the other necessary EAAs. Although leucine is described as the most effective of the three BCAAs in stimulating muscle protein synthesis, isoleucine and valine also play a significant role. In fact, their deficiency inhibits muscle growth despite the presence of leucine. Furthermore, exclusive supplementation with high doses of BCAAs can generate competitive transport mechanisms with other AAs, reducing their availability and consequently limiting biosynthesis [105]. Therefore, to counteract the hypercatabolic state, impaired protein synthesis, and reduced intracellular AAs transport observed in metabolic disorders, it is essential to provide a supplement containing all EAAs in appropriate proportions. Indeed, AAs depletion in the TME not only restricts local availability but also drives a systemic reduction, contributing to muscle wasting, impaired protein metabolism, and broader metabolic dysfunction, with significant negative consequences for patient outcomes.

Previously, in a randomized phase II study involving 25 cachectic subjects with advanced cancer, the administration of 8 g/day of EAAs for 8 weeks was associated with the maintenance of residual MM, a significant increase in strength and total serum albumin, with a decrease in blood levels of ROS [146]. Given that, unlike healthy cells, tumor cells do not exhibit a clear distinction between EAAs and NEAAs and are reliant on NEAAs, the administration of all EAAs in a stoichiometric ratio may represent a promising strategy. This approach could alter the EAA/NEAA ratio balance, thereby modulating the TME, inhibiting tumor progression, preserving MM, and improving systemic outcomes, as demonstrated in vivo experimental models [34,35].

Although the available findings on EAAs supplementation in cancer patients are encouraging, it should be noted that most clinical evidence derives from phase II studies involving relatively small patient cohorts. Large-scale randomized clinical trials are still lacking, warranting caution in interpreting and applying these results in clinical practice. Moreover, responses to EAAs supplementation may vary among different tumor types, influenced by factors such as dosage, composition of the mixture, and duration of administration.

## 7. Conclusions

Both quality and quantity of EAAs (EAA/NEAA ratio) introduced by nutrition would regulate an efficient balance between protein synthesis and protein turnover [105,147]. Based on current evidence, we propose that continuous supplementation of cancer patients with a complete mixture of EAAs, formulated in a stoichiometric ratio aligned with human cellular requirements, may enhance protein synthesis while shifting the typical dietary EAA/NEAA ratio toward values ≥1. This metabolic shift could impose stress on tumor cells, eliciting an epigenetic response marked by increased protein synthesis and heightened ATP consumption. However, in the context of excessive EAAs and limited availability of NEAAs, efficient protein assembly in cancer cells would be impaired. To compensate, tumor cells may activate hyper-autophagy and de novo NEAAs synthesis, further elevating ATP demand and leading to increased ROS production and oxidative stress. These metabolic disturbances may suppress mTORC1 signaling, arrest protein synthesis, induce apoptosis, and ultimately promote cancer cell death.

In summary, current evidence supports the concept that supplementation with a complete mixture of EAAs may serve as a valuable adjunct to standard cancer therapies. This strategy has the potential to correct cancer-associated protein imbalances, improve patients’ quality of life, and create a metabolic environment unfavorable to tumor progression. Nevertheless, well-designed clinical trials are needed to fully clarify the therapeutic efficacy and safety of EAAs supplementation in oncology.

## Data Availability

Not applicable.

## Abbreviations

The following abbreviations are used in this manuscript:

Arg-1	Arginase 1
ASS1	Arginosuccinate synthase
BCAA	Branched-chain amino acids
CRC	Colorectal cancer
EAAs	Essential amino acids
eNOS	Endothelial nitric oxide synthase
IDO1	Indoleamine 2,3 dioxygenase
MM	Muscle mass
NEAA	Non-essential amino acids
ROS	Reactive oxygen species
TME	Tumor microenvironment
